# Case report: Safety and efficacy of adalimumab in treating difficult-to-treat rheumatoid arthritis in a human immunodeficiency virus-positive patient, one year follow-up

**DOI:** 10.3389/fimmu.2022.942642

**Published:** 2022-08-03

**Authors:** Jing-Wen Chen, Guo-Shu Deng, Wen-Shuang Zhang, Ming-Ying Zhang, Tong Guan, Qiang Xu

**Affiliations:** ^1^ The First Clinical Medical College, Guangzhou University of Chinese Medicine, Guangzhou, China; ^2^ Department of Rheumatology, The First Affiliated Hospital of Guangzhou University of Chinese Medicine, Guangzhou, China

**Keywords:** rheumatoid arthritis, adalimumab, TNF, human immunodeficiency virus, AIDS

## Abstract

Rheumatoid arthritis (RA) is a joint-disabling inflammatory disease associated with the pathology of synovitis. Some patients with RA are difficult to treat, using disease-modifying anti-rheumatic drugs (DMARDs). Biology and targeted synthetic DMARDs (b/tsDMARDs) are options for patients with RA. Acquired immunodeficiency syndrome (AIDS) is an infectious disease caused by the human immunodeficiency virus (HIV). Adalimumab is an anti-tumor necrosis factor therapy commonly used in patients with RA. However, there are no reports or related data on patients with RA-HIV/AIDS treated with adalimumab are available. In this report, we described the first successful case of a 60-year-old HIV-positive woman with difficult-to-treat RA treated with ADA after being screened for hepatitis virus, latent tuberculosis (LTBI), and other infections. She contracted HIV from sexual exposure while on adalimumab therapy. As the patient was resistant to first-line DMARDs, she continued adalimumab along with the initiation of highly active antiretroviral therapy (HAART). The patient was treated with adalimumab therapy for a year; her CD4+ lymphocyte count was normal, HIV-1 RNA decreased, and no new infections were triggered. The patient achieved clinical remission of RA. In conclusion, adalimumab is a safe option for patients with RA-HIV and may slow the progression of HIV infection. Furthermore, HAART has the potential to reduce joint pain and fatigue in patients with difficult-to-treat RA.

**Conclusions:**

Adalimumab is a safe option for patients with RA-HIV, and may slow down the progression of HIV infection. The HAART therapy has the potential to reduce joint pain and fatigue in patients with difficult-to-treat RA.

## Introduction

Rheumatoid arthritis (RA) is a joint-disabling inflammatory disease that is associated with the pathology of synovitis, and some patients are difficult to treat, using disease-modifying anti-rheumatic drugs (DMARDs). Treatment options for RA have expanded with the advent of biological and targeted synthetic DMARDs (b/tsDMARDs) (1). In case of failure of DMARDs treatment, physicians may choose b/tsDMARDs for patients with difficult-to-treat RA. However, according to the British Society for Rheumatology, the risk of latent tuberculosis (LTBI) and hepatitis triggered by bDMARDs should not be ignored (2).

According to data from the United Nations Program on HIV/AIDS, approximately 37.7 million people worldwide are living with HIV in 2020, of which approximately 10.2 million were untreated, and approximately 1.5 million are newly infected. Furthermore, approximately 680,000 people died from AIDS in 2020. Approximately 7,000 new HIV infections in both adults and children are reported worldwide every day. Highly active antiretroviral therapy (HAART) has dramatically altered the development and transmission of HIV/AIDS. In contrast, the worldwide prevalence of RA is approximately 0.5–1% (1). This high prevalence means that RA and HIV may co-occur in some individuals. This high prevalence indicates that RA and HIV may co-occur in some individuals. The Veterans Aging Cohort Study showed that among patients positive for rheumatoid factor (RF), 14 of 1,521 patients with HIV (0.9%) could be classified as having incident RA, compared to 145 of 4,020 individuals without HIV (3.6%) (3). However, only few reports on patients with RA and HIV are available, and the treatment is challenging.

TNF-α is involved in the pathogenesis of HIV-1 infection, and serum concentrations of TNF-a have been shown to increase with the progression of HIV-1. TNF-α may contribute to disease progression. Therefore, TNF-α inhibitors are theoretically attractive for use in the setting of HIV infection (4, 5). TNF-α is a critical upstream molecule in joint inflammation and plays a role in balancing bone destruction and bone formation in RA (1). TNF-α also plays a key role in the pathogenesis of HIV/AIDS (6). Some HIV proteins mimics the molecular function associated with TNF-α, which may activate downstream pathways associated with the TNF-α signaling pathway and may be a therapeutic target for patients with RA-HIV. Adalimumab is an anti-TNF therapy that is commonly used in patients with RA. However, no reports or related data are available on patients with RA-HIV/AIDS treated with adalimumab. In this report, we described the first successful case of a 60-year-old, HIV-positive woman with difficult-to-treat RA who was treated with ADA after screening for hepatitis virus, LTBI, and other infections. During ADA treatment, she contracted HIV from sexual exposure and showed elevated HIV-RNA replication and decline in CD4+ lymphocyte counts during the early stages of infection (**Table 1**). As the patient was resistant to first-line DMARDs, she continued to receive adalimumab along with the initiation of HAART. We closely monitored her CD4+ lymphocyte counts and HIV-RNA levels but did not trigger new infections. This patient achieved clinical remission of RA. At the same time, we also found that HAART relieved the symptoms of joint pain and fatigue in RA patients.

**Table 1:** Tests for quantitation of HIV RNA (Started HAART:11/5/2021).

Date	Result	Normal range	Method
16/4/2021	1.31E+5	<1.00E+2 IU/mL	NucliSens Esay Q
11/1/2022	2.90E+1	<20 copies/mL	Amplicor Cobas

## Case description

A 60-year-old woman presented with fatigue and weight loss, along with pain in the proximal interphalangeal joints of both hands, wrists, elbows, shoulders, knees, and metatarsophalangeal joints. She was diagnosed with RA at a local clinic six months ago. Her symptoms were not relieved with six months of continuous treatment of DMARDs, and the patient had fever. When the patient referred to our hospital, she experienced severe pain in the small- and medium-sized joints throughout the body and significant swelling in the interphalangeal joints of all painful joints. She described her joints as having a burning sensation, accompanied by significant morning stiffness. She was positive for antinuclear antibody and negative for RF. Additionally, she had a high titer of anti-cyclic citrullinated peptide antibody, an erythrocyte sedimentation rate of 48 mm/h, and a CRP level of 13.3 mg/L (**Table 2**). Using the 28-Joint Disease Activity Scale (DAS28), her diagnosis of RA was confirmed with a disease activity score of 6.04, suggesting a high degree of disease activity.

**Table 2:** Baseline of laboratory tests results.

Test name	Normal range	Results	Method
White blood cells	4.0-10.0×10^9^ /L	5.61	Flow Cytometry
Neutrophils	2.0-7.5×10^9^ /L	2.45	Flow Cytometry
Eosinophils	0.05-0.3×10^9^ /L	0.11	Flow Cytometry
Lymphocytes	1.6-4.0×10^9^ /L	2.48	Flow Cytometry
Hemoglobin (HGB)	110-150g/L	115	Colorimetry
Platelet	100-300×10^9^ /L	259	Sheath flow
Aspartate aminotranspherase (AST)	<=32	18	Rate
Alanine aminotransferase (ALT)	<=33	12	Rate
Creatinine (Cr)	41-73μmol/L	55	Oxydase reaction
Serum uric acid (UA)	143-357μmol/L	335	uric acid enzyme
Albumin (ALB)	40.0-55.0g/L	40.4	Bromocresol green
C-reactive protein (CRP)	0-8mg/L	13.2	Scatter turbidimetry
Erythrocyte sedimentation rate (ESR)	0-20mm/h	48	Instrumental
Rheumatic factor (RF)	0-20.0IU/ML	<20.0	Scatter turbidimetry
Anti-citrullinated protein antibody (Anti-CCP)	0-5U/ML	>200	Chemoluminescence
Complement 3(C3)	0.79-1.52g/L	0.947	Scatter turbidimetry
Complement 3(C4)	0.16-0.38g/L	0.333	Scatter turbidimetry
Antinuclear antibody (ANA)	Negative	Positive	Indirect immunofluorescence
Anti-Smith protein antibodies (Anti-SM)	Negative	Negative	Chemoluminescence
anti-SSA antibody	Negative	Negative	Chemoluminescence
anti-SSB antibody	Negative	Negative	Chemoluminescence
Tuberculosis test (T-SPOT)	Negative	Negative	Immunofluorescence
Hepatitis c antibody	Negative	Negative	Chemoluminescence
Hepatitis B surface antigen (HBsAg)	Negative	Negative	Chemoluminescence
Antistreptolysin O (ASO)	0-116IU/ML	<25.0	Scatter turbidimetry

**Figure 1:**
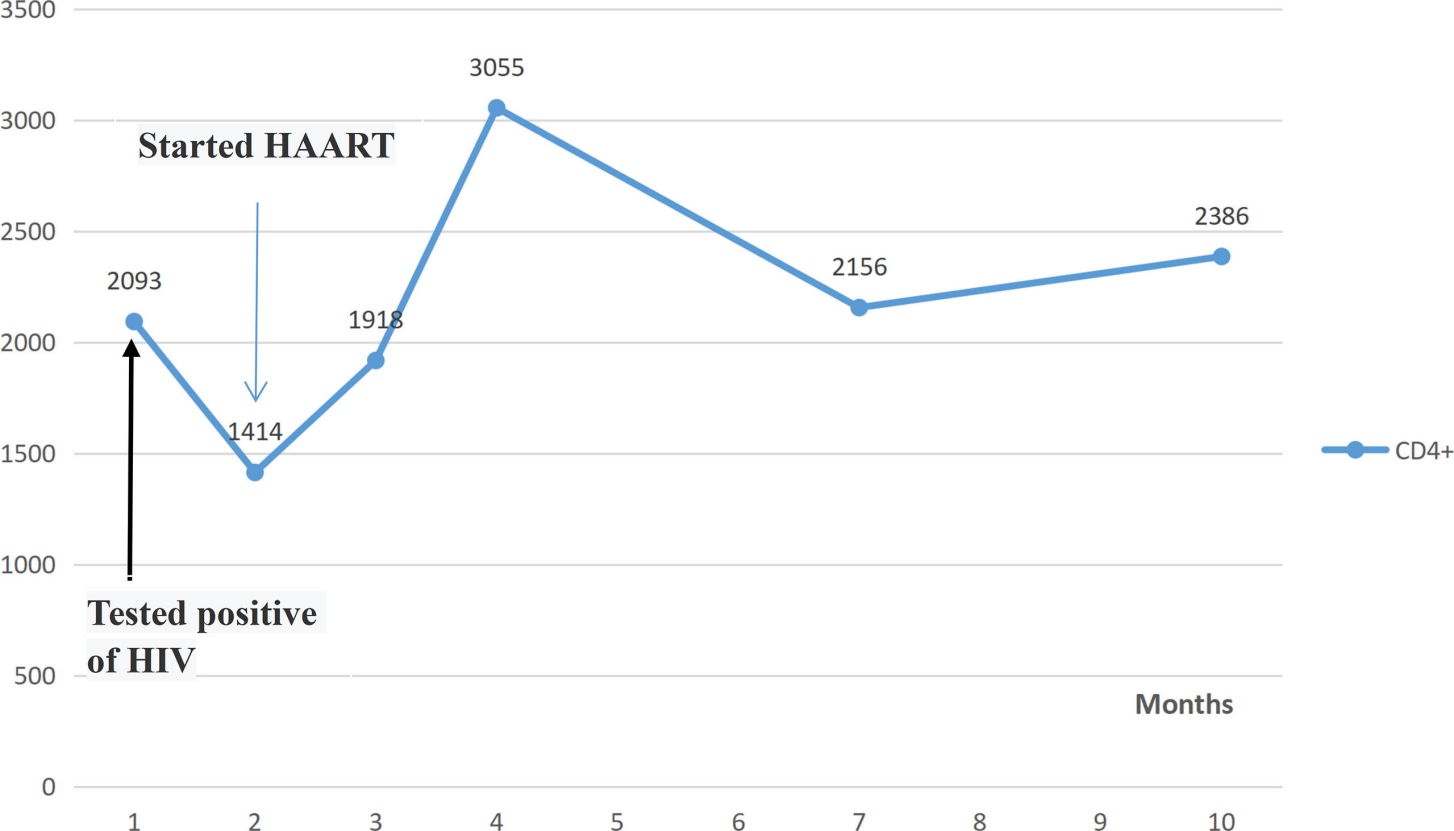
CD4+ lymphocytes counts during the treatment of an HIV-positive patient with difficult-to-treat RA with using adalimumab. Normal CD4+ lymphocyte count: 1488-4483U/μL.

Next, we initially administered methotrexate (MTX), tofacitinib, and etoricoxib because of the ineffectiveness of multiple DMARDs, for which the patient experienced insignificant joint pain relief. The patient experienced vomiting after MTX administration. For several reasons, the patient refused low-dose steroids to relieve pain. As this patient was resistant to first-line DMARDs, we initiated adalimumab (40 mg subcutaneously every other week) (Hisun Biopharmaceutical Co., Ltd.) therapy after screening the patient for hepatitis virus infection, LTBI, and other infectious conditions according to the British Society for Rheumatology DMARDs safety guidelines. Compared to DMARDs, the patient responded better to adalimumab therapy. Unfortunately, the patient had a concealed history of a sexual partner with HIV/AIDS. However, her DAS28 score decreased to 4.9 after two weeks of ADA treatment, and symptoms such as joints pain and weakness were significantly relieved. Nonetheless, after RA was in control, HIV was detected in her blood, with a quantitative HIV-1 RNA test revealing values of 1.31E+5 IU/mL, accompanied by a decreased CD4+ lymphocyte count. Considering that she had difficult-to-treat RA combined with HIV/AIDS, her swollen and painful joints were uncontrolled. In addition, the patient was ineffective against DMARDs. Steroids may have accelerated the replication of HIV-RNA and induce other infections. After discussion with the patient, adalimumab therapy was continued, and HAART was initiated. HIV-1 RNA viral replication was controlled, and the CD4+ lymphocyte count continued to increase (**Figure 1**). After one year of ADA therapy, LTBI and other infections did not occur. She had no fever, and her fatigue had significantly improved. Her case was considered as RA remission, with a DAS28 score of 1.40.

## Discussion

Here we have a 60-year-old woman with difficult-to-treat RA, who was exposed to HIV during ADA treatment. In this case report, our patient did not respond to conventional DMARDs, and her synovial inflammation was highly active. However, she responded well to ADA therapy. Since the patient had subsequent HIV/AIDS infection with concomitant RA activity, the risk of induction of HIV or other infections using steroids was significant. Therefore, the patient was not suitable for steroids. The 2021 ACR guidelines (7) also emphasize the importance of reducing steroid use.

The abnormal immune response in HIV infection indicates that HIV virus infection is also closely related to the occurrence of rheumatism (8). Rheumatological manifestations (RM) are very common in HIV patients. A retro review of the published literature was performed by searching the PubMed and MEDLINE databases using the key words “rheumatologic” and “HIV” (**Table 3**). In 1989, Kaye (13) described that the Reiter syndrome, reactive arthritis, polymyositis, and the sicca syndrome may herald the onset of clinically evident HIV infection. These diseases and others may also occur in patients with full-blown AIDS.A cross-sectional study (10) in India included 75 patients with HIV infection, of whom 35 had RM (46.67%). Arthralgia was the most common manifestation of HIV infection, with 20 cases (26.67%), followed by myalgia in 14 cases (18.67%) and arthritis in 10 cases (13.33%). 14 patients (18.66%) developed myalgia, 10 patients (13.33%) had arthritis, two patients had HIV-related arthritis, one patient had oligoarthritis of the left knee and ankle, and another patient had symmetrical polyarthritis of the wrist and ankle, which was alleviated at 4-6 weeks. Rheumatoid factor (RF) and Human Leukocyte Antigen-B27 (HLA-B27) were negative, and the sacroiliac joint was normal and non-invasive. The cohort study in Taiwan (15) analyzed a total of 22327 patients newly diagnosed with HIV during 2000–2012.Compared with the general population, patients with HIV have a higher risk of developing Sjogren’s syndrome(SS), psoriasis, systemic lupus erythematosus(SLE), autoimmune hemolytic anemia and uveitis, but a lower risk of ankylosing spondyloarthritis (AS). A review in Phoenix, Arizona showed that 113 out of 2996 HIV patients (3.8%) were found to have a rheumatologic condition. The common autoimmune disease was psoriasis(1%),rheumatoid arthritis (0.23%), psoriatic arthritis(0.2%) and systemic lupus erythematosus(0.2%) (9). Amit Gajera and Susan Kais (11) reported a 35-year-old female with HIV not on HAART was diagnosed whit HIV polyarteritis nodosa-like vasculitis presenting as chronic abdominal pain. Anthony G et al (16) described seven black African boys with HIV infection. Arthritis was polyarticular in six boys and pauciarticular in one. Four had intermediate uveitis and three boys had non-granulomatous anterior uveitis. Ocular complications included cataract, cystoid macular edema, hypotony, pupillary membranes, band keratopathy, posterior synechiae and optic disk edema. A research on Congo involved 3042 patients among whom 306 (10%) were positive for HIV, 220 (7.2%) presented with a rheumatologic manifestation, and 158 (71.8%) with HIV related arthritis (12). In order to assess the frequency of RM at pediatric human HIV infection, Martínezrojano H et al described 26 HIV-infected children, which RM were identified in 5 (19.2%),involved biphasic Raynaud’s syndrome, necrosing vasculitis, lip necrosis and livedo reticularis, knee arthalgias, vasculitis, and septic arthritis of the ankle (14).

**Table 3:** Reported cases of HIV and rheumatologic manifestations.

Author	Country or Area	Year of publication	Type of the study	Rheumatologic manifestations of HIV infection
Yung-Feng Yen et al.	Taiwan, China	2016	Review	Sjögren Syndrome, psoriasis, SLE, autoimmune haemolytic anaemia and uveitis.
Parperis K et al. (9)	Phoenix, Arizona, American	2019	Review	Psoriasis (1%), rheumatoid arthritis (0.23%), psoriatic arthritis (0.2%) and systemic lupus erythematosus (0.2%).
Renu Saigal et al. (10)	India	2020	Review	Spondyloarthritis (8%), psoriatic arthritis (1.67%). HIV associated arthritis (2.67%), septic arthritis, rheumatoid arthritis, vasculitis, and diffuse infifiltrative lymphocytic syndrome were seen in 1.33%
Amit Gajera & Susan Kais (11)	/	2009	Case report	A 35-year-old female with HIV not on HAART was diagnosed whit HIV polyarteritis nodosa-like vasculitis presenting as chronic abdominal pain.
Anthony G et al.	South Africa	2008	Review	Arthritis was polyarticular in 6 patients and pauciarticular in 1. Four patients had intermediate uveitis and 3 patients had nongranulomatous anterior uveitis.
Ntsiba.H et al. (12)	Congo	2007	Review	7.2% presented with a rheumatologic manifestation, and 158 (71.8%) with HIV related arthritis.
Kaye BR (13)	/	1989	Meta-analysis	The Reiter syndrome, reactive arthritis, polymyositis, and the sicca syndrome may herald the onset of clinically evident HIV infection. These diseases and others may also occur in patients with full-blown AIDS.
Martínezrojano Het al. (14)	Mexico	2001	Review	Rheumatologic manifestations were identified in 19.2%, involved biphasic Raynaud's syndrome, necrosing vasculitis, lip necrosis and livedo reticularis, knee arthalgias, vasculitis, and septic arthritis of the ankle.

Although HIV-related rheumatic immune diseases have been reported in different regions, recommendations for treatment are still rare. The drug therapy of RA, SS in HIV positive is worth exploring. How to balance the safety and efficacy of drug therapy of rheumatoid arthritis in the setting of HIV-positive still need more clinical experiments.

The use of anti-TNF therapy in patients with HIV infections has also been reported. Cepeda et al. (17) treated eight HIV-positive patients with anti-TNF: among which two patients had RA, three had psoriatic arthritis, one had undifferentiated spondyloarthritis, one had reactive arthritis, and one had ankylosing spondylitis. Treatment with anti-TNF therapy is a viable alternative for patients with advanced RA who fail to respond to standard therapy. In a multicenter study of psoriatic arthritis (18), three HIV-positive patients were subjected to co-administration of HAART and adalimumab therapy. All patients showed improvement in the psoriasis area and severity index. None of these patients experienced worsening of HIV infection during the course of adalimumab treatment. All patients showed improvement in the psoriasis area and severity index (PASI). None of these patients experienced any worsening of HIV over the course of ADA treatment. TNF-α is involved in the viral replication and pathogenesis of HIV. TNF- α can promote the expression of HIV through nuclear factor kappa (NFkB) signal pathway (8). Stephanie M (19)through the analysis of clinical trials and case series, show that tumor necrosis factor inhibitors can improve HIV/AIDS patients with fewer complications. But the CD4+ cardinality still needs to be monitored more closely. Although there are some positive reactions in HIV-infected patients treated with biological agents, there are also negative results. A RA-HIV patient had been treated with etanercept, but developed septic shock two weeks after receiving influenza vaccination. The condition deteriorated rapidly and developed into disseminated intravascular coagulation. She was eventually intubated, dialyzed and survived (20). In 2010, the National Psoriasis Foundation Medical Board (21) only recommended the cautious use of anti-TNF-α inhibitors in closely monitored patients with HIV and very refractory psoriasis or debilitating psoriatic arthritis. Physicians should screen for LTBI, tumors, hepatitis B and hepatitis C, as well as other serious infectious diseases before initiating treatment with a TNF-α inhibitor, but b/tsDMARDs for patients with HIV-positive at the same time, there are still no new recommendations in the 2021 American Rheumatology Association Rheumatoid Arthritis guidelines (7).

Adalimumab is an immunosuppressive drug and one of the inhibitors of TNF-α. It has been approved for a medical treatment since 2003 for RA, juvenile idiopathic arthritis (JIA), ankylosing spondylitis (AS), psoriasis (PSA), psoriasis (Ps), hidradenitis suppurativa (HS), uveitis and Crohn’s disease (CD). Adalimumab is usually recommended for patients who have failed to respond to other treatments. As a biologic agent, the safety of adalimumab deserves clinical attention. A systematic review and meta-analysis of randomized controlled trials (22) showed that an increase in infection among those that received adalimumab normal dosage (RR: 1.13, 95% CI: 1.04-1.23), and in patients with psoriasis (RR: 1.13, 95% CI: 1.00-1.35) and rheumatoid arthritis (RR: 1.23, 95% CI: 1.06-1.41), but not in those that received high doses and other criteria. In the meta-regressions, intervention duration was not related to changes in incidence risk. Infection is the most common adverse effect of adalimumab treatment. A randomized study (23) included 523 patients treated with adalimumab plus methotrexate(MTX). 386 (74%) of 523 patients reported treatment-emergent adverse events. There was a single case of disseminated tuberculosis, six opportunistic infection and two candidiasis and one aspergillosis in the adalimumab group. Three deaths were reported, one was a sudden death, one death caused by pneumonia, and one by bronchopulmonary aspergillosis. A 27-year-old female was detected by the interferon-γ release assay as LTBI, started isoniazid treatment (300 mg/day) 3 weeks before starting adalimumab and maintained this for 6 months. After 45 weeks of adalimumab therapy. However, she was diagnosed with Disseminated Tuberculosis (24). Retrospective series of eight patients showed that treatment with anti-TNF-a is a viable alternative in HIV patients without advanced disease with associated rheumatic diseases refractory to standard therapy. Four patients were additionally treated with infliximab and/or adalimumab after not achieving a satisfactory clinical response with etanercept. There were no clinically significant adverse effects. All patients’ CD4+ counts and HIV viral load levels remained stable (17). There are few case reports of adalimumab in HIV infection as well as a lack of long-term follow-up. In our case report, this RA-HIV patient was treated with adalimumab throughout, and HIV-RNA and CD4+ counts remained stable during the one year follow-up without the new infection.

TNF-α is an important inflammatory pathway in the pathogenesis of RA, and its levels in HIV-infected patients are also suggestive of HIV/AIDS pathogenesis. Elevated TNF-α levels are associated with increased viral replication, a subsequent increase in HIV-RNA and decrease in CD4+ lymphocyte counts (25). Anti-TNF therapy may be a safer therapeutic target for patients with RA-HIV. Theoretically, the role of TNF-α in HIV/AIDS suggests that anti-TNF-α therapy may contribute to treatment. However, given the important role of TNF-α in host defense against infection, anti-TNF therapy increases susceptibility to infection, such as *Mycobacterium tuberculosis* and atypicalum. Therefore, patients with RA-HIV should focus more on LTBI.

Previously, we emphasized that major infections, such as hepatitis virus and LTBI, should be screened in RA patients using DMARDs and b/tsDMARDs; however, data on RA-HIV patients are scarce, and case reports or clinical data on the application of biologic therapy in such patients are rare. As HAART is clinically used, the survival of HIV patients is prolonged, and the number of patients presenting with chronic rheumatologic disease is increasing. Treatment of HIV/AIDS patients with comorbid immune diseases is challenging. To our knowledge, this is the first case report of RA-HIV treated with adalimumab. During the one-year follow-up observation, continued use of anti-TNF therapy did not increase HIV-RNA levels, CD4+ lymphocyte counts remained stable, and showed a possibility of slowing down the progression of HIV infection.

Previous epidemiological studies have shown that the prevalence of autoimmune diseases (such as SS) decreases significantly after the initiation of HAART.A cohort study in Taiwan (15) also showed that HIV-infected patients treated with HAART had a higher risk of psoriasis, autoimmune hemolytic anemia and uveitis, but a lower risk of RA and AS. DMcGonagle (26) reported a patients with HIV-SpA who received routine antirheumatic therapy, and arthritis was still worsening, but significantly improved after HAART. At the same time, CD4+ lymphocyte count increased significantly. Here we showed a difficult-to-treat RA patient in an HIV-positive was successfully treated with adalimumab. We also found that HAART relieved the symptoms of joint pain and fatigue in RA patients. Further clinical responses should be evaluated. With the successful application in this patient, anti-TNF therapy may be a new therapeutic direction for the treatment of patients with HIV combined with other arthritis, which needs to be supported by more data. Furthermore, we observed significant improvements in fatigue symptoms after HAART, which may have the potential to reduce joint pain and fatigue in patients with difficult-to-treat RA. In the future, anti-TNF therapy in HIV may be reserved for new patients who have failed other treatments and are well controlled under HAART.

## Conclusion

This case study shows that adalimumab is a safe option for patients with RA-HIV, may slow the progression of HIV infection, and has the potential to reduce joint pain and fatigue in patients with difficult-to-treat RA.

## Data availability statement

The original contributions presented in the study are included in the article/supplementary material. Further inquiries can be directed to the corresponding authors.

## Ethics statement

Written informed consent was obtained from the patient for the publication of any potentially identifiable images or data included in this article.

## Author contributions

J-WC, G-SD, and W-SZ contributed to the data collection and drafting of the manuscript. M-YZ helped to revise the manuscript. TG and QX conceived the study, and revised the manuscript. All authors contributed to the article and approved the submitted version.

## Conflict of interest

The authors declare that the research was conducted in the absence of any commercial or financial relationships that could be construed as a potential conflict of interest.

## Publisher’s note

All claims expressed in this article are solely those of the authors and do not necessarily represent those of their affiliated organizations, or those of the publisher, the editors and the reviewers. Any product that may be evaluated in this article, or claim that may be made by its manufacturer, is not guaranteed or endorsed by the publisher.
